# A qualitative study on the role of cultural background in patients' perspectives on rehabilitation

**DOI:** 10.1186/1471-2474-13-5

**Published:** 2012-01-23

**Authors:** Mandy Scheermesser, Stefan Bachmann, Astrid Schämann, Peter Oesch, Jan Kool

**Affiliations:** 1 Zurich University of Applied Sciences, School of Health Professions, Institute of Physiotherapy, Technikumstrasse 71, 8401 Winterthur, Switzerland; 2 Department of Rheumatology, Rehabilitation Centre, 7317 Valens, Switzerland; 3 Department of Geriatrics, Inselspital, University of Bern Hospital, 3010 Bern, Switzerland

## Abstract

**Background:**

Low back pain (LBP) is one of the major concerns in health care. In Switzerland, musculoskeletal problems represent the third largest illness group with 9.4 million consultations per year. The return to work rate is increased by an active treatment program and saves societal costs. However, results after rehabilitation are generally poorer in patients with a Southeast European cultural background than in other patients. This qualitative research about the rehabilitation of patients with LBP and a Southeast European cultural background, therefore, explores possible barriers to successful rehabilitation.

**Methods:**

We used a triangulation of methods combining three qualitative methods of data collection: 13 semi-structured in-depth interviews with patients who have a Southeast European cultural background and live in Switzerland, five semi-structured in-depth interviews and two focus groups with health professionals, and a literature review. Between June and December 2008, we recruited participants at a Rehabilitation Centre in the German-speaking part of Switzerland.

**Results:**

To cope with pain, patients prefer passive strategies, which are not in line with recommended coping strategies. Moreover, the families of patients tend to support passive behaviour and reduce the autonomy of patients. Health professionals and researchers propagate active strategies including activity in the presence of pain, yet patients do not consider psychological factors contributing to LBP. The views of physicians and health professionals are in line with research evidence demonstrating the importance of psychosocial factors for LBP. Treatment goals focusing on increasing daily activities and return to work are not well understood by patients partly due to communication problems, which is something that patients and health professionals are aware of. Additional barriers to returning to work are caused by poor job satisfaction and other work-related factors.

**Conclusions:**

LBP rehabilitation can be improved by addressing the following points. Early management of LBP should be activity-centred instead of pain-centred. It is mandatory to implement return to work management early, including return to adapted work, to improve rehabilitation for patients. Rehabilitation has to start when patients have been off work for three months. Using interpreters more frequently would improve communication between health professionals and patients, and reduce misunderstandings about treatment procedures. Special emphasis must be put on the process of goal-formulation by spending more time with patients in order to identify barriers to goal attainment. Information on the return to work process should also include the financial aspects of unemployment and disability.

## Background

Low back pain (LBP) is one of the major concerns in health care. In Switzerland musculoskeletal problems represent the third largest illness group with 9.4 million consultations per year [1]. Of these 9.4 million consultations, approximately 30% are related to LBP. LBP also has the highest prevalence of medical conditions in the working-age population in Switzerland with 8% of all working-age women and 13% of all working-age men affected in a 4 week period in 2002 [2]. Total costs of LBP in Switzerland are estimated at 7.4 billion Euros. Direct medical costs amount to 3.4 billion Euros corresponding to 6.7% of total Swiss health care expenditures, while indirect costs are estimated at 4.0 billion Euros [3]. In the majority of patients LBP is non-specific. Evidence suggests that for less than 15% of individuals LBP can be assigned to a specific LBP category such as nerve root compression, vertebral fracture, tumour, infection and definite instability [4].

Chronic LBP, normally defined as a non-specific low-back pain persisting more than 12 weeks [5] is one of the most frequent reasons for persistent disability and inability to work. The expenses of the Swiss Disability Insurance increased by 215% between 1990 and 2005, and approximately 20% of disability pensions in 2008 were due to musculoskeletal diseases among which LBP played a predominant role [6]. Regarding the socioeconomic impact of LBP, efficient management has high priority. Previous research compared a function-centred treatment (FCT) to a pain-centred treatment (PCT). The FCT emphasized activity despite pain through work simulation, strength, endurance, and cardiovascular-training. PCT in contrast emphasized pain reduction trough passive and active mobilization, strength training and a mini back school with education and exercise. After one year, patients with FCT had significantly more workdays (mean, 118; median, 39.5; interquartile range [IQR], 0-198) than patients with PCT (mean, 74; median, 0; IQR, 0-160) [7,8]. The authors therefore suggest that the return to work rate may be increased by an active treatment program[7,8] and may be cost saving [9]. Results related to the returning to work rate were poorer (Odds Ratio (OR): 0.30; Confidence Interval (CI) 95% = 0.13-0.73) compared with patients born in Switzerland [10]. This qualitative research explores possible barriers to return to work that have not been identified and addressed adequately so far.

The purpose of this study was to understand the experience of patients with chronic LBP and a Southeast European cultural background in multidisciplinary rehabilitation programs. To meet these aims, two questions were identified: 1) what factors do patients in multidisciplinary rehabilitation programs for LBP perceive to be important for acceptance or participation, and 2) are patients' perspectives similar to those of health professionals and scientific literature?

## Methods

### Study design

**Figure 1:**
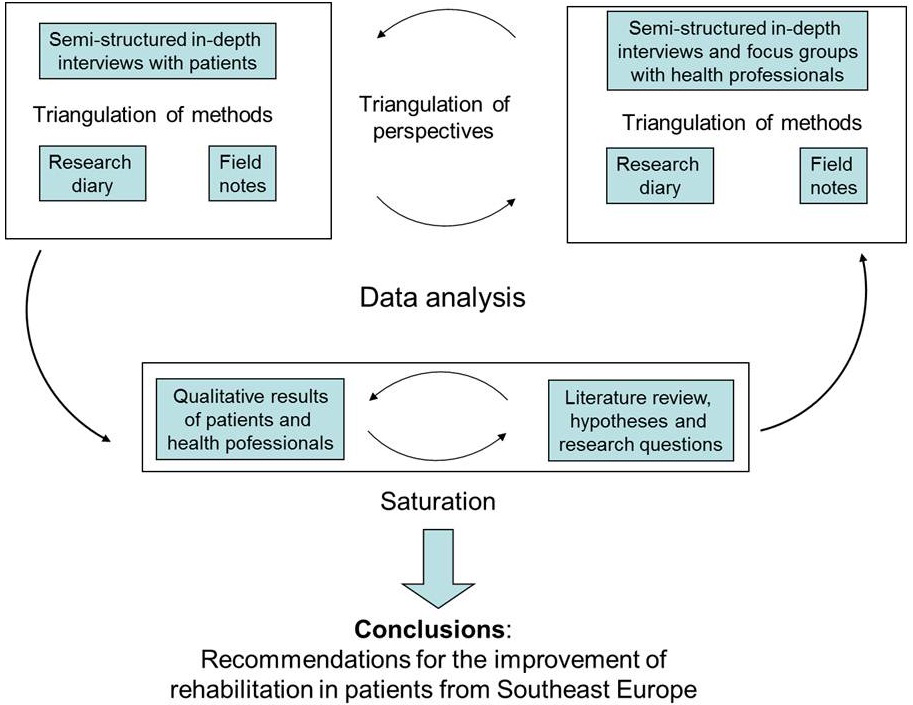
**Design of the study analysing semi-structured in-depth interviews with patients and health professionals, focus groups with health professionals and the results from a literature review**.

Figure 1 gives an overview of the study's methods. We based the design and the procedure on the six quality criteria of qualitative research [11]: 1) documentation of the procedure, 2) coverage of the interpretation by argumentation, 3) systematic procedure, 4) closeness to the object of research, 5) validation by communication, and 6) triangulation of methods. The last criteria was considered [12] combining three qualitative methods of data collection: 1) semi-structured in-depth interviews with patients, 2) semi-structured in-depth interviews and focus groups with health professionals, and 3) a literature review. The attributes of the procedure are described according the COREQ consolidated criteria for reporting qualitative research [13] (see Additional file 1). The study was approved by the ethical committee of the canton St. Gallen, Switzerland (EKSG 03/035).

### Samples

Patients were recruited at the Rehabilitation Centre Clinic Valens in the German-speaking part of Switzerland. A researcher at the rehabilitation clinic contacted face-to-face potential new subjects. Selection was guided by purposive sampling to get insight to experiences of immigrants from Southeast European cultures attending a function-centred rehabilitation program. Patients therefore were included by the following criteria: a) participation in a function-centred rehabilitation program, b) migration background, c) Southeast European cultural background, and d) Serbo-Croatian mother tongue.

Health professionals involved in the function-centred rehabilitation of patients with chronic LBP were asked to participate. We arranged one focus group (six persons) with physical and one focus group (six persons) with occupational therapists. Five individual interviews with other health professionals including physicians, psychiatrists, social workers and nurses were conducted.

### Data collection

The semi-structured in-depth interviews and focus groups used a system of categories as a guideline based on theoretical and empirical literature and focused on the different topics displayed in Table 1. The interview guide was pre-tested for usability by means of a pilot interview. The duration of interviews and focus groups was between 40 and 95 minutes. We repeated neither interviews nor focus groups. Interviews were audio-recorded, translated, transcribed verbatim, and stored using a pseudonym. Field notes were not taken. As the transcriptions were produced verbatim, they were not returned to participants for comments or corrections.

**Table 1:** General topics of semi-structured in-depth interviews and focus groups

Topics covered in semi-structured in-depth interviews with patients

• Treatment expectations

• Perceptions of reality

• Communication and information about diagnosis and treatment

• Occupation and disability

• Cultural background

Topics covered in focus groups and interviews with health professionals

• Work experience

• Function-centered rehabilitation

• Communication

• Recommendations for treatment

The semi-structured in-depth interviews with patients were always conducted by the same female psychologist, speaking the patients' native language as first language. The interviewer was independent of the clinic and of the research project, and she was not expected to take field notes. No one else attended the interviews. Patients were welcomed, and the interviewer introduced herself and explained the circumstances of the research project and the purpose to get insight into patient's needs and aspects valuable for improvement of the rehabilitation program.

Focus groups with health professionals and individual interviews with other health professionals were conducted by the first author of this study, a female social scientist.

### Data analysis

Transcripts of the interviews and focus groups were analyzed using qualitative content analysis as described by Mayring [14]. The aim of this analysis is to reduce the material by maintaining the constitutive contents and to create a manageable corpus through abstraction, not changing the image of the basic material [14]. The qualitative data analysis software Atlas.ti [15] was used for coding the data by two independent scientists. The authors read the transcripts several times in order to get a sense of the focus groups and interviews in their entirety. In a second step, information that reflected the influence of cultural factors on rehabilitation was marked and the coding-system was developed in an iterative process: After coding about 30% of the material, the coding system was adapted in terms of entirety, for improving reliability. After closing the coding-system, the whole material again was recoded where required. Then we combined related codes in code families (subcategories and leading categories). The coding tree is available in the online supplement materials (see Additional file 2). Subcategories with comparable content were summarized to leading categories. Participants didn't provide feedback on the findings.

### Systematic literature search

We collected relevant information from the identified literature. The literature search was done in the data bases Web of Science and Pub Med using the following search strategy for the literature focusing on function-centred rehabilitation:

(Title (TI) = low back pain) AND (Topic search (TS) = rehabilitation) AND ((TS = sick leave) OR (TS = return to work) OR (TS = activity) OR (TS = functional impact) OR (TS = physical activity) OR (TS = work retention) OR (TS = disability pension) OR (TS = improvement) OR (working ability)).

**Figure 2:**
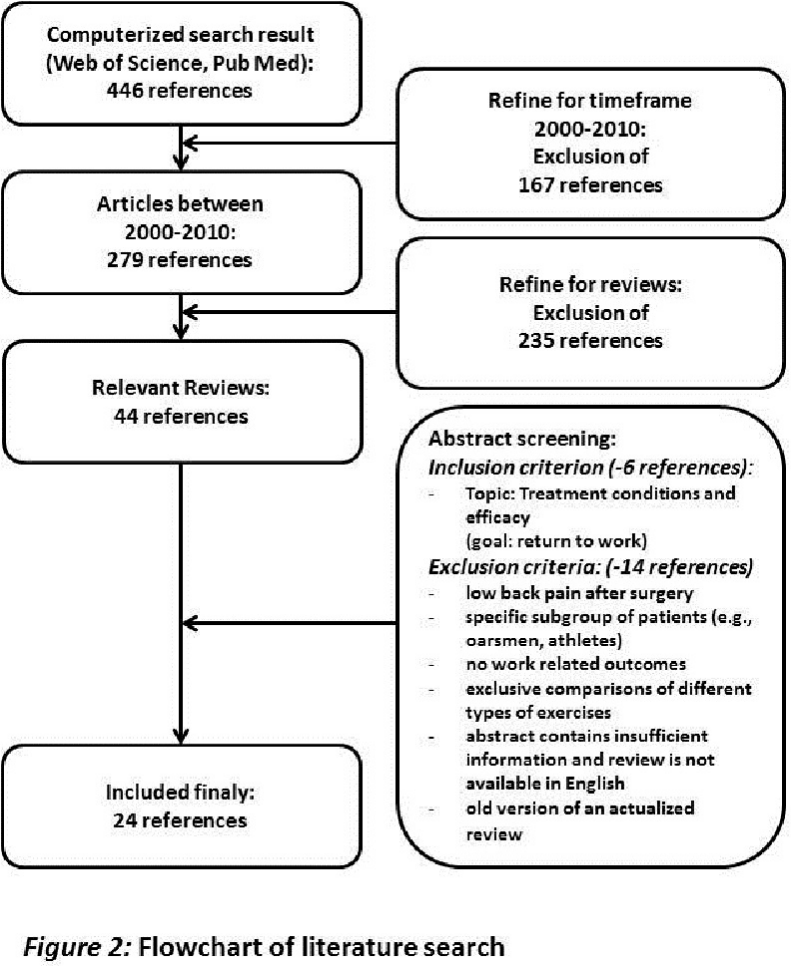
**Flowchart of literature search**.

The search results and the in- and exclusion criteria of the literature are displayed in Figure 2. 24 reviews about rehabilitation in low back pain, regarding work-related outcomes, were included.

For the literature about associations of low back pain, culture, rehabilitation and work, we searched for: (TS = Low Back Pain) AND (TS = Cross-Cultural Comparison) AND ((TS = Yugoslavia) OR (TS = Serbia) OR (TS = Macedonia (Republic)) OR (TS = Bosnia) OR (TS = Croatia) (TS = Slovenia) OR (TS = Montenegro)) AND (TS = Rehabilitation).

As this search retrieved no results, we moved on to a grey literature search via the Swiss National Library and the Administration of Swiss Government and via reference tracking.

We searched for themes and titles in the Swiss National Library using the following keywords: (migrants OR migration OR refugees OR culture) AND (pain OR health OR rehabilitation) AND (work OR job). 21 references resulted from this search. Titles and abstracts were screened, and references were excluded if they applied the concept of culture to specific subgroups with local backgrounds (e.g., youth culture, culture of alcohol addicts) or if they relied on cultural comparisons between Western Europe and the USA. One dissertation on health conceptions and behaviours of migrants from Kosovo remained. Eight references have been drawn from the reference list of this dissertation, as they relied on health and migration in Switzerland and Germany. Two references have been included at a researchers' of the Administration of the Swiss Government suggestion. Overall, eleven articles and books have been included containing information on the topic of health (specifically low back pain), culture, rehabilitation and work.

The identified literature was compared with our empirical data as displayed in the results section. Finally, we formulated on the basis of our data possible improvements for the practice of function-centred rehabilitation and outcomes in patients with a Southeast European cultural background.

## Results

### Characteristics of the patients

Between June and December 2008, 30 patients fulfilled the inclusion criteria and were invited to participate in the study. 13 patients (aged 38-60 years) gave their informed consent. The duration of the interviews was between 40 and 95 minutes. Table 2 contains further information about the patients in this study. The majority of patients had 8 years of education with the exception of one patient who had 12 years of education. 5 out of 13 patients had a professional qualification. One participant had been lived separated from his family in Croatia for 22 years, and has been visiting his family twice a month.

**Table 2:** Characteristics of patients with LBP

Characteristics	Men (n = 9)	Women (n = 4)
age (mean)	52	48

professional education	5	0

country of birth		

Serbia	2	1

Croatia	3	1

Bosnia	2	1

Macedonia	1	0

Kosovo	0	1

duration of stay in Switzerland (years, mean)	24.5 years	16 years

married	9/9	4/4

financial support of children	6	2

duration of back pain (years, mean)	7 years	3.5 years

work load (max load to be lifted at work)		

light (< 10 kg)	2	1

medium-heavy (10-20 kg)	3	3

heavy-very heavy (> 20 kg)	4	0

The patients' current or last jobs were: machine operator (5), cleaner (2), kitchen assistant (2), welder (1), metal worker (1), refuse collector (1), and car mechanic (1). The majority of respondents had decided to live in Switzerland for financial reasons. Two had arrived as refugees during the war in former Yugoslavia. One patient grew up in Yugoslavia while his parents had been living in Switzerland and followed them later. Eight patients said the expectations they had before coming to live in Switzerland had been fulfilled. For two patients, expectations were partially fulfilled: "In some ways yes, the economic standard is higher in Switzerland and cars are not as expensive." Three patients were disappointed: "All in all, my expectations have not been fulfilled."

### Content analysis of interviews and triangulation with research evidence

Content analysis of semi-structured in-depth interviews with patients and health professionals revealed six categories of themes: A) management of back pain, B) function-centred rehabilitation, C) goals of rehabilitation, D) communication, E) family, and F) psychological aspects. Table 3 summarizes the categories and their sub-categories as well as the main conclusions from the semi-structured in-depth interviews of patients and health professionals. We performed a triangulation of results from three sources of information: 1) interviews with patients, 2) interviews with health professionals, and 3) research evidence. We formulated interim conclusions for each sub-category of themes.

**Table 3:** Categories of analysis and main results for patients and health professionals

Categories:	Sub-categories:
A. management of back pain	• previous management of back pain • cause and meaning of back pain • rehabilitation by activity and exercise

B. function-centered rehabilitation	• patient expectations of treatment • patients' coping strategies • patients' assessment of treatment

C. goals of rehabilitation	• goals of patients' and health professionals' • return to work

D. communication	• language barriers • talking at across purposes

E. family	• family support • differences of family support between women and men

F. barriers to rehabilitation	• psychological aspects • financial concerns

### A. Management of back pain

#### Previous management of back pain

##### Patients' perspective

Patient had suffered from LBP for an average of 3.5 years (women) and 7 years (men). Previous treatment had been pain-centred and passive, using rest, sick-listing, medication, passive physiotherapy and psychotherapy. One patient stated: "I took different drugs. I don't know which medications I have not yet taken." (patient 2). Many patients were trapped in a vicious circle of increasing pain and consumption of drugs. Patients did not take responsibility for their health instead passing all responsibility over to the health care system. They also preferred passive treatments including medication and rest, and did not understand why they should increase activity in the presence of pain, even though health professionals seek to increase patients' activity, coping and involvement.

##### Health professional's perspective

One health professional noted: "Patients seem to be filled with fear of harming their back and making everything worse. The physician is the expert and knows what is wrong. Patients don't feel confident enough to take responsibility." Health professionals also felt that patients do not have the power to get active and change their situation: "Patients think the physician is the expert and will 'fix' the problem. They do not consider their own contribution towards improvement. Patients who suffer from pain find it hard to understand that they have to be active."

##### Perspective of literature review

Research concludes that the most effective treatments for disability with the goal of returning to work include exercise or multidisciplinary rehabilitation [5,8,16-27]. Passive treatments, on the other hand, have not been demonstrated to induce long-term improvements of work activity. While the onset of centralization of pain and behavioural changes occur within the first two months of absence from work [28], multidisciplinary rehabilitation should start much earlier and is recommended if a patient cannot return to work within six weeks of the onset of LBP [20,27]. Research identifies contradictory treatment preferences in patients and health professionals. Patients see themselves as a victim of their complaints, and expect therapy or help from outside [29]. Patients also expect solutions from the health care system [30]. Contributory factors are not only associated with their cultural background but also with political and economic factors [31]. Compared to the Germans, Austrians and the French, Southeast European migrants exercise less [32]. This difference is more pronounced in women.

##### Interim conclusion 1

Current pain-centred practice reported by many patients is not in line with treatment guidelines that emphasize a return to normal activities, exercise and earlier multidisciplinary rehabilitation. There is a considerable mismatch between patients' treatment preferences and those of health professionals and evidence-based recommendations. To cope with pain, most of patients predominantly use passive strategies. However, several health professionals and researchers propagate active strategies including activity in the presence of pain. Although this message is communicated to patients, patients avoid activities that increase pain.

#### Cause and meaning of back pain

##### Patients' perspective

Almost all patients were convinced that the origin of their LBP was musculoskeletal even if they had repeatedly been informed of negative findings in x-rays and Magnetic Resonance Imaging (MRI). One patient stated: "I have pain so there must be a physical cause" (patient 2). Some patients reported that diagnostic procedures did not identify a somatic diagnosis for their LBP (patient 4). Other patients attributed their complaints and disability to irrelevant radiological findings such as degenerative changes of the lumbar spine. In summary, it seemed that patients were upset that their pain could not be substantiated and therefore expressed their suffering by limping, pain postures, moaning, self-limitation and consumption of pharmaceuticals. While some patients received controversial information from different physicians, others attributed LBP to findings even if their physician emphasized that these were not associated with the patients' complaints or that the degree of degeneration was "normal". All patients reported sleep-disturbances, depressed mood or fear of increasing disability. In spite of these symptoms, they denied a possible contribution of psychological factors to LBP. One patient, for example, argued: "This is 100% not the cause of pain" (patient 3). Many women did not associate chronic back pain with double stress from work and household: "I do not think that I am over-extended. All the women I know are working full-time and do all the housework. Their families need the money." (patient 6).

##### Health professional's perspective

Several health professionals reported that many patients incorrectly focus on mechanical causes of LBP. It seems that, because of language and communication problems, patients frequently misunderstand diagnostic and management information. A physician reported: "Patients have their own curious explanations about their back, the cause of pain and which is the best treatment. It is not easy to change these misconceptions." A psychiatrist recalled that patients did not accept psychological aspects to be related to LBP. Psychological issues stay within the family. Physical causes of LBP are easier to understand and accept and are visible, for example on MRI.

##### Perspective of literature review

Research shows that people with a Southeast European cultural background tend to have different concepts of body and mind than Western European people [33]. Other literature demonstrates that psychological factors are related to LBP [19,20,34,35]. Patients suffering from headache or neck pain accept psychological causes whereas patients with back or leg pain do not [36]. Patients may also reject psychological causes because these suggest that "the pain does not really exist" and, last but not least, because psychological causes are stigmatized [29,33,37].

##### Interim conclusion 2

Several patients do not accept that psychological factors contribute to LBP. The views of physicians and health professionals are in line with research evidence demonstrating the importance of psychosocial factors for LBP.

#### Rehabilitation by activity and exercise

##### Patients' perspective

Patient 5 said that physicians in the former Yugoslavia offer 'special methods' like 'recovering a pinched nerve through bioenergy'. He recalled: 'Natural healers fix arms, legs and backs'. Almost all patients prefer the medical care provided in Switzerland, however, there are crucial differences in the treatment of LBP in Switzerland and Southeast Europe. Consequently, Southeast European patients in Switzerland expected passive treatments like medication, relaxation, rest, massage, hot packs and hydrotherapy. They were not familiar with active treatment and considered it "illogical" and "counterproductive": "In Switzerland medication is prescribed only to go back to work. But heavy work aggravates the pain." (patient 10) Moreover, patients felt that in their home country, they had more possibilities for adapted work: "It is up to you if you want to stay at home or work as much as you can." (patient 11).

##### Health professional's perspective

Some participating health professionals thought that some patients from Southeast Europe demonstrated stronger behavioural responses related to pain, more frequently attributed pain to severe disease and took more medical advice.

##### Perspective of literature review

Research identifies pronounced differences in the treatment of LBP in Switzerland and Southeast Europe. In Southeast European countries, common treatments include the use of galenicals, visiting medicine prophets and attending ritual practices. Patients with a Southeast European background have a different understanding of disease and "health seeking behaviour" [33,38]. As a consequence, expectations of physicians and patients regarding the diagnosis and treatment of LBP differ significantly. In fact, LBP is perceived almost as a completely different disease in Switzerland and in Southeast Europe [33].

##### Interim conclusion 3

Almost all interviewed patients prefer Western medical treatment over traditional treatment. Health care differences are obvious and should be considered as a factor that may reduce treatment effectiveness.

### B. Function-centred rehabilitation

#### Patients' expectations of treatment

##### Patients' perspective

Majority of patients expressed high treatment expectations: "I expect fast help, to be cured, healthy and pain free" (patient 2). Some patients expected that physicians "take away the pain" and that "my back pain gets better automatically". They expected more pain-centred passive treatment, e.g. massage, hot packs and relaxation in the pool. They disliked active training, training on their own, walking and swimming. Only two patients were satisfied with function-centred treatment, most patients experienced little or no improvement to their LBP; one even felt worse and more tired.

##### Health professional's perspective

However, several health professionals felt that increasing activity was a realistic goal and that the patients' expectations of total pain relief were unrealistic.

##### Perspective of literature review

Literature suggests that patients have very high expectations of the physician. Physicians are regarded as having a comprehensive knowledge of diagnoses and therapies [29]. Patients expect physicians to treat and cure them without a substantial contribution of their own [31].

##### Interim conclusion 4

Lots of patients have unrealistically high outcome expectations. They also expect passive pain-centred treatments, whereas health professionals and literature focus on function-centred treatment. Moreover, the treatment goals are not well understood by the patients partly due to communication problems. (c.f. category 4 "communication").

#### Patients' coping strategies

##### Patients' perspective

A large number of patients reported that they had "designed" their own strategies to reduce pain, such as taking pain medication, adopting a pain free position and self-massage: "I figured it out myself. If I have aches and pains I have to lie or to walk a little bit. This position [...] reduces back pain so it must be good." (patient 1).

9 out of 13 respondents agreed, that "activity has a positive impact on health". Three patients said they considered activity to be important because physicians and therapists told them so: "If I have to go for a long time, it isn't good for me. But I know that I have to do this. Every physician says that." (patient 1). Only one patient agreed with the statements "exercise increased my pain" and "exercise isn't good, but I do it for my body." Although one patient thought activity caused serious inflammation, the major opinion was that exercise was good but did not improve back pain. However, exercise was felt to cause and aggravate pain if certain limits were exceeded: "I like to walk slowly, but can't go upstairs! If I walk a long distance, back pain is aggravated."(patient 6). "To walk downhill is easy, uphill is difficult. I can't and I won't, because I'm not a hiker."(patient 1). Some patients felt compelled to do exercise: "Therapists say I have to do exercise but I can't. [...] I feel horrible pain. I get nervous if I feel pain."(patient 6). Another patient stated: "I can walk 100 meters but I need short breaks and to sit down. Therapists tell me to walk without a break."(patient 10).

##### Health professional's perspective

Some health professionals reported that because many patients have passive coping strategies to deal with pain, it was not easy to persuade patients to try other strategies. Health professionals also assumed that patients did not understand the information and did not believe in new strategies that they recommended: "Patients often do not appreciate the content of therapy." One health professional noted: "It doesn't make sense for patients to take a walk. They prefer taking drugs."

##### Perspective of literature review

Research emphasizes the importance of coping strategies [5,8,16,17,22,28,39-41]. Cognitive behavioural therapy and function-centred rehabilitation include coping strategies [16,19,42].

##### Interim conclusion 5

Patients' coping strategies are not in line with recommended coping strategies.

#### Patients' assessment of treatment

##### Patients' perspective

A function-centred rehabilitation program includes weekly consultations with a physician. However, half of the patients would like to have seen their physician more frequently: "I have seen my physician only three times - just on the ward rounds. [...] I had no extensive conversation."(patient 2).

##### Health professional's perspective

Interviewed physicians stated that their role was to coordinate multidisciplinary treatment and those weekly visits were sufficient.

##### Perspective of literature review

Research suggests that frequent physician visits can be a social legitimization of pain [29]. Physician-patient-conversation displays "communication between two different cultures" [33]. Exercise or activity is regarded as an essential part of behavioural treatment [8]. This suggests that simply increasing the number of physician's consultations will not improve outcome.

##### Interim conclusion 6

There is a discrepancy between patients' expectations and physicians' opinions regarding the required frequency of consultations during rehabilitation. Research evidence does not support increasing the number of consultations. As long as patients consider physicians as having authority, physicians can use this authority to increase their impact on patients' compliance.

### C. Goals of rehabilitation

#### Goals of patients' and health professionals'

##### Patients' perspective

Most patients said they wanted to "be more active" and "return to work" but only under the condition that pain was relieved first.

##### Health professional's perspective

Health professionals propagate increasing activity and accepting an initial pain increase. They formulate patient-specific function-centred treatments towards long-term improvement in daily activities including return to work.

##### Perspective of literature review

Important rehabilitation goals are the development of coping strategies, increasing walking distance and velocity, and improving physical capacity related to maintaining work postures, manual object handling or lifting [5,8]. These goals are supported by research recommending multidisciplinary rehabilitation where the use of these goals is a key element [5,16-18,20,23,27,39].

##### Interim conclusion 7

At first sight both patients and health professionals agree on the treatment goal of returning to work but patients and health professionals have contrary views on the strategy to reach this goal. These opposing opinions about treatment strategies contribute to the general problem of talking at cross-purposes and differences in outcome expectations.

#### Return to work

##### Patients' perspective

All patients stated that they are "willing to return to work immediately", although only under certain circumstances, for example: "if I am pain-free". One patient reported: "but with this disease I can't do my current job or any other job at 100%." (patient 8). The seemingly high readiness to return to work could be determined by social desirability. Many patients seemed to think health professionals considered them to be malingerers if they did not say they wanted to return to work. In addition, return to work was the primary goal of rehabilitation: "I want to, I have to, but I can't return to work."(patient 2). Probably to demonstrate their willingness to return to work, several patients tried to return to work. Somatic and psychological problems were described by a patient who tried to return to work: "I wanted to work for two or three hours but it was too hard [...] my psychological constitution did not allow me to go on [...]. I felt useless, and I thought I was just a disturbance." (patient 4).

##### Health professional's perspective

Interviewed health professionals observed that majority of patients said they wanted to return to work only if they were "completely healthy and pain-free during activity". A physician reported that all patients would return to work if their body was "immaculate", again, "but as long as this situation was not achieved, patients considered a return to work impossibility."

##### Perspective of literature review

Research has repeatedly demonstrated that low job satisfaction and adverse working conditions are risk factors for a longer duration of absence from work [20,34,35,43]. Most of these factors are associated with the cultural background of patients but they have no causal relationship. Reviews confirm that an optimal rehabilitation requires close cooperation with insurers and employers: Thus, it is possible to influence working conditions and facilitate return to work (e.g. with adapted workplace design, treatment by a work physician, and implementation of prevention programs) [20,21,28,34,35,44].

##### Interim conclusion 8

Social desirability contributes to patients stating that they want to return to work. Poor job satisfaction and other work-related factors constitute barriers to returning to work.

### D. Communication

#### Language barriers

##### Patients' perspective

The majority of patients experienced communication problems due to language barriers: "I cannot speak German very well. I understand about 40 per cent which is not enough to understand the physicians." (patient 9). Most patients declared that they had problems understanding "medical language": "I don't understand medical terms." (patient 1). Although support from interpreters is increasingly used, patients advocated more support.

##### Health professional's perspective

Almost all health professionals confirmed the existence of language barriers, partly reinforced by low educational levels and a lack of belief in self-management. Misunderstanding reduces compliance with advice and the continuation of active therapy at home: "Firstly, lots of patients do not understand the language, and secondly, they do not believe in self-management."

##### Perspective of literature review

Research identifies linguistic and cultural communication problems between patients and health professionals. On the one hand, patients do not speak German well enough. On the other hand, they have a different cultural background with regard to physician-patient interaction [32,33,45] and are suspicious towards diagnostic information [36,46]. It is essential to inform patients about the diagnosis, therapy and the activities to be followed in detail. Communication between patient and health professionals should use general language. Others have suggested the use of symbols to support communication with patients [33].

##### Interim conclusion 9

Language barriers constitute an important factor in hindering efficient treatment. Although interpreters are increasingly used in health care, most communication between health professionals and patients takes place without such assistance and is, therefore, prone to misunderstandings and is limited in its potential for deeper discussion and more extensive explanations using general language.

#### Talking at across purposes

##### Patients' perspective

Majority of patients described talking at cross-purposes with health professionals: "I told the physician I can't do my work because my back hurts. He said it hurts because I have weak muscles and haven't worked for the last seven months." (patient 2). Another patient reported: "He [the physician] looks at the monitor while typing something on the computer. Thus, it is certainly not surprising that we talked past each other." (patient 3). Several patients thought that physicians did not understand them: "I have the feeling that they don't trust me and think that I am simulating back pain. But they don't feel my aches and pains." (patient 1).

##### Health professional's perspective

Many interviewed health professionals also felt that misunderstandings occur frequently: "Not only do we have language barriers; we also talk at cross purposes."

##### Perspective of literature review

Research shows that aspects of language and culture are strongly related and impair communication between patients and health professionals [32,33,45]. Characteristics of good clinical communication and interaction between physicians and patients with LBP are: a) that patients feel that they are being taken seriously, b) that they are given an understandable explanation of their pain, c) pain-centred care is applied, and d) that patients are well informed about treatment procedures. Results show an important potential for enhancement of clinical communication with patients.

##### Interim conclusion 10

Communication problems impair the rehabilitation process. Both interviewed patients and health professionals are aware of communication problems due to cultural differences in communication.

### E. Family

#### Family support

##### Patients' perspective

Lots of patients with LBP are looked after by their family members who relieve them of physical activities. Many patients receive more attention and are encouraged to take rest. The positive feeling of being supported is counteracted by the negative feeling of uselessness, associated with being off work. During inpatient rehabilitation, almost all patients missed their families. They were not used to being separated from their family for several weeks at a time. Main part of patients wished "to go home to their family as soon as possible". In the rehabilitation centre patients did not take centre stage and were "one among many". This may have contributed to patient's complaints of insufficient individual treatment, as they felt alone and misunderstood. Accordingly, their motivation for treatment was generally poor.

##### Health professional's perspective

Several health professionals thought that family members frequently relieved patients of their duties, took over responsibility and reduced their autonomy: "Patients have a clear gain from illness and hardly any chance of changing their situation".

##### Perspective of literature review

Research suggests that the extended family is the most important frame of reference for patients. Paternalistic family structures determine lifestyle. The family gives security and cares for the sick, not the state, and the state should not interfere with the family [30]. During his or her sickness, the family member is the focus of the family [33]. Family support represents a substantial gain from illness.

##### Interim conclusion 11

Differences in the roles of the family and the state in Switzerland and Southeast Europe are one of the most obvious cultural factors identified in our study. Patients' families support passive behaviour and reduce patients' autonomy. Paternalistic family structures are important and may be difficult to influence. Nevertheless, cooperation with the patient's family might facilitate successful rehabilitation and return to work.

#### Differences of family support between women and men

##### Patients' perspective

All female respondents carried a double workload of job and household. One woman noted: "In addition to my 100% job, I carry full responsibility for the housework, look after my husband and children... like every woman does." (patient 6). Female patients did not have enough free time for themselves: "I go to work, do the housework, prepare a meal and afterwards go to the fitness studio - terribly tired and in pain!" (patient 6).

##### Health professional's perspective

Interviewed health professionals pointed out that double stress is a specific problem for women: "Most women additionally care for their children and husband. Hoping to live up to all possible expectations, women are under considerable strain." If women get sick, they often do not find the way back to normality. For many women it is not relevant to formulate goals for partial restitution of activity: "Either do everything, or do nothing." One therapist reported: "Some female patients have it twice as hard [as men in general] because their family does not accept their sudden illness". Several participating health professionals observed that many families have a traditional distribution of roles: "Men do not help women with housekeeping, particularly men of the older generation."

##### Perspective of literature review

Research shows that roles in Southeast European families show more pronounced paternalistic patterns [30]. Women alone are responsible for the household [36] and psychological distress is more severe in women from the former Yugoslavia [32]. This is essential for rehabilitation from chronic pain, as reviews point out that low social support, low decision latitude, high psychosocial work demands, more social isolation and female gender are risk factors for the chance of returning to work [34,43].

##### Interim conclusion 12

Many families relieve patients of their duties, take over responsibilities and reduce their autonomy. Almost all women have multiple responsibilities including work, household and care of their children and their husband. Women with LBP are less supported by their families.

### F. Barriers to rehabilitation

#### Psychological aspects

##### Patients' perspective

Majority of interviewed patients feared painful activities, doing something wrong, harming their back and having to have surgery. Many patients had too little self-confidence to take control of their own future and found it difficult to accept help from others: "People from my country are not used to others [i.e. the rehabilitation team] trying to help. Instead of facing my future, I look away like a little child... without self-confidence... not knowing where to go." (patient 4).

##### Health professional's perspective

Some health professionals confirmed that participating patients feared disability and a future dependency on wheelchairs. Many patients had worked with LBP for years and feared that work would damage their back. As a result, several patients may require more time to regain confidence in their back.

##### Perspective of literature review

Hasani [30] describes how disability in patients with a Southeast European cultural background is associated with psychological problems. Accordingly, overestimation and uncertainty about disability cause fear in patients and their families. Thus, patients are uncertain and fear negative consequences for their families.

##### Interim conclusion 13

Management of LBP-related disability could be improved by clearer information about disability and therapy.

#### Financial concerns

##### Patients' perspective

Some patients who were unemployed on grounds of ill health had serious concerns about their financial future. They complained of sleep disorders and mental problems: "Without medication I can't sleep. I don't know what is going to happen."(patient 11).

##### Health professional's perspective

Participating health professionals confirmed that many patients were under considerable strain: "for men, in particular it is very stressful because family members have high expectations. They are financially responsible for their family in Switzerland and additionally for family members in their homeland. They feel useless without a job".

##### Perspective of literature review

Research shows that immigrants in Switzerland suffering from LBP are employed in sectors with a higher risk of disability. Because these people generally have a lower level of education, they have hardly any chance of finding lighter work [47]. Low socioeconomic status is a risk factor for chronic illness and disability [33].

##### Interim conclusion 14

Financial security and socioeconomic status are important risk factors for disability in most patients and families with a Southeast European cultural background.

The interim conclusions will be discussed in the next section. Overall conclusions are formulated in the conclusions section.

## Discussion

The focus of this qualitative study was on modifiable aspects of rehabilitation that may improve treatment outcome. Many patients with a Southeast European cultural background do not accept that psychological aspects may contribute to LBP. Communication problems impairing the rehabilitation process are related to limited German language ability, as well as cultural differences in communication. Most patients have high treatment expectations and are disappointed when these are not fulfilled. Moreover, patients' goals do not match those of health professionals because patients prefer pain-centred treatment and reduce activities that cause pain. Although avoiding activities that increase pain initially relieves pain and makes patients "feel good", the consequence is that activity tolerance decreases and pain is eventually felt at lower intensities of activity. Patients are, thus, caught in a vicious circle, also known as the "feel good trap". As a result, activity should be gradually increased in spite of pain because improving activity tolerance decreases pain in the long term. This message is repeatedly communicated to patients but, regardless of language and culture, many patients with chronic pain find it difficult to understand, accept and adopt.

Based on current evidence, multidisciplinary rehabilitation aiming at increasing activity is the treatment of choice, even though treatment is accompanied by discomfort and even a pain increase. Discomfort accompanies treatment of chronic pain and many other treatments aiming at changing behaviour such as losing weight by changing eating habits, stopping smoking, and changing one's lifestyle to improve physical fitness. Avoiding necessary efforts and short-term discomfort are frequent barriers to successful treatment.

If none of the patients were to benefit from movement in spite of pain, treatment would seem unethical. The majority of patients who increasingly use active coping strategies manage to decrease disability and return to work. This supports the applied treatment concept and justifies its use.

Many issues identified in this study may, after all, not only be cultural. Many factors are associated with social background [32]. To give two examples, both lower education and heavy manual work are associated with poorer outcome, independent of cultural background [7]. Some researchers also questioned whether cultural background is predictive for outcome and concluded that social background is more important than culture [48]. Despite these considerations, it seems plausible that cultural factors exist.

Several barriers to better rehabilitation outcomes are more pronounced in patients from Southeast Europe, for instance language problems hindering communication between patients and health professionals, patients' treatment expectations focusing on help by physicians and health professionals, and passive coping strategies. For many patients from Southeast Europe, their family and relatives play an important role. During inpatient rehabilitation, patients miss their families, as they are not used to being separated from them for several weeks.

### Study strengths and limitations

Strengths of this study include semi-structured in-depth interviews that were conducted by a native speaker. The triangulation of results from the semi-structured in-depth interviews with patients and health professionals, and scientific literature increases the reliability and validity of the results. The results are highly relevant as they aim at inducing a change in rehabilitation practice.

Weaknesses of the study are that we conducted only 13 patient interviews. Saturation may not have been reached and information may have been missed. Furthermore, we only contacted patients in one rehabilitation centre and only patients from the region of the former Yugoslavia. Therefore, it might be possible that the information would be different, if patients were interviewed in other rehabilitation centres. The conclusions drawn from our research are strictly only valid for patients from the former Yugoslavia. People from other countries and ethnic groups may have other ideas and beliefs regarding treatments. Our findings do not allow causal interpretations between cultural factors and treatment outcome for two reasons. Firstly, this is a qualitative study that was designed to develop hypotheses on how the treatment of patients from Southeast Europe can be improved. Secondly, the cross-sectional design of this study is inadequate for causal inferences as it is unknown whether the proposed causal factor actually preceded the effect. Finally, a comparison group is lacking.

It is important to implement return to work management early, including return to adapted work to improve rehabilitation in patients with a South European cultural background. Special emphasis must be given to the process of formulating goals by spending more time with patients to identify barriers to goal attainment. We further propose the involvement of patients' families and increased use of interpreters. Information on the return to work process should also include the financial aspects of unemployment and disability. During rehabilitation, more attention should be given to patients developing outlooks for the future.

## Conclusions

LBP rehabilitation may be improved by addressing the following points. Early management of LBP should be activity-centred instead of pain-centred. It is mandatory to implement return to work management early, including return to adapted work to improve rehabilitation for patients. Rehabilitation has to start when patients have been off work for three months. Using interpreters more frequently could improve communication between health professionals and patients, and reduce misunderstandings about treatment procedures. Putting special emphasis on the process of goal-formulation and spending more time with patients in order to identify barriers to goal attainment should enhance rehabilitation progress. Information on the return to work process should also include financial aspects of unemployment and disability.

## Competing interests

The authors declare that they have no competing interests.

## Authors' contributions

All authors were involved in the preparation of this study and in writing the grant proposal for the Swiss National Science Foundation. MS planned and analysed the semi-structured in-depth with patients and health professionals. MS performed, planned and analysed focus groups with health professionals. JK and MS performed the literature review. All authors read and approved the final manuscript.

## Pre-publication history

The pre-publication history for this paper can be accessed here:

http://www.biomedcentral.com/1471-2474/13/5/prepub

## Supplementary Material

Additional file 1 **Consolidated criteria for reporting qualitative studies (COREQ): 32-item checklist**. Click here for file

Additional file 2 **Coding tree**. Click here for file
