# Supplementary material for: A qualitative study on the role of cultural background in patients' perspectives on rehabilitation
Source: BMC Musculoskelet Disord. 2012 Jan 23;13:5. doi: 10.1186/1471-2474-13-5 (PMC3398320; doi:10.1186/1471-2474-13-5)
Supplement: Additional file 1 — Consolidated criteria for reporting qualitative studies (COREQ): 32-item checklist. [file 1471-2474-13-5-S1.DOC]

**Consolidated criteria for reporting qualitative studies (COREQ): 32-item checklist**

Developed from:

Tong A, Sainsbury P, Craig J. Consolidated criteria for reporting qualitative research (COREQ): a 32-item checklist for interviews and focus groups. *International Journal for Quality in Health Care*. 2007. Volume 19, Number 6: pp. 349 – 357

| **No. Item** | **Guide questions/description** | **Reported on Page #** |
| --- | --- | --- |
| **Domain 1: Research team and reﬂexivity** | | |
| *Personal Characteristics* |  |  |
| 1. Interviewer/facilitator | Which author/s conducted the interview or focus group? | MS=focus groups with health Professionals  Mirjana Canjuga (MC)=Interviews with patients (see acknowledgement) |
| 2. Credentials | What were the researcher’s credentials? E.g. PhD, MD | MS= social scientist, MSc, PhD Student MC= psychologist, MSc, PhD Student |
| 3. Occupation | What was their occupation at the time of the study? | MS=researcher at ZHAW, MC=researcher at ETH Zürich |
| 4. Gender | Was the researcher male or female? | p. 6, LN 18 and p. 7, LN 2 |
| 5. Experience and training | What experience or training did the researcher have? | Qualitative studies are part of theirs university education, research experience at different institutions for years |
| *Relationship with participants* |  |  |
| 6. Relationship established | Was a relationship established prior to study commencement? | Yes, because previous studies were conducted in Rehabilitation Centre Valens, see Reference 7,8 |
| 7. Participant knowledge of the interviewer | What did the participants know about the researcher? e.g. personal goals, reasons for doing the research | p. 6, LN 21-23 |
| 8. Interviewer characteristics | What characteristics were reported about the interviewer/facilitator? e.g. Bias, assumptions, reasons and interests in the research topic | Independent research team, experiences in research topic cultural background, MC has a Serbo-Croatian cultural background |
| **Domain 2: study design** | | |
| *Theoretical framework* |  |  |
| 9. Methodological orientation and Theory | What methodological orientation was stated to underpin the study? e.g. grounded theory, discourse analysis, ethnography, phenomenology, content analysis | p. 7, LN 4-5 |
| *Participant selection* |  |  |
| 10. Sampling | How were participants selected? e.g. purposive, convenience, consecutive, snowball | p. 5, LN 22-23, p. 6, LN 1-9 |
| 11. Method of approach | How were participants approached? e.g. face-to-face, telephone, mail, email | p. 5, LN 22-23, p. 6, LN 6-7 |
| 12. Sample size | How many participants were in the study? | p. 9, LN 4 |
| 13. Non-participation | How many people refused to participate or dropped out? Reasons? | p. 8, LN 3-4 |
| *Setting* |  |  |
| 14. Setting of data collection | Where was the data collected? e.g. home, clinic, workplace | p. 5, LN 22-23 |
| 15. Presence of non-participants | Was anyone else present besides the participants and researchers? | p. 6, LN 20-21 |
| 16. Description of sample | What are the important characteristics of the sample? e.g. demographic data, date | p. 6, LN 1-9 |
| *Data collection* |  |  |
| 17. Interview guide | Were questions, prompts, guides provided by the authors? Was it pilot tested? | p. 6, LN 13 |
| 18. Repeat interviews | Were repeat interviews carried out? If yes, how many? | p. 6, LN, 14-15 |
| 19. Audio/visual recording | Did the research use audio or visual recording to collect the data? | p. 6, LN 14 |
| 20. Field notes | Were ﬁeld notes made during and/or after the interview or focus group? | p. 6, LN 14-15 |
| 21. Duration | What was the duration of the interviews or focus group? | p. 6, LN 13 |
| 22. Data saturation | Was data saturation discussed? | p. 24, LN 14-15 |
| 23. Transcripts returned | Were transcripts returned to participants for comment and/or correction? | p. 6, LN 16 |
| **Domain 3: analysis and ﬁndings** | | |
| *Data analysis* |  |  |
| 24. Number of data coders | How many data coders coded the data? | p. 7, LN 7-8 |
| 25. Description of the coding tree | Did authors provide a description of the coding tree? | p. 7, LN 14 |
| 26. Derivation of themes | Were themes identiﬁed in advance or derived from the data? | p. 7, LN 9-14 |
| 27. Software | What software, if applicable, was used to manage the data? | p. 7, LN 7 |
| 28. Participant checking | Did participants provide feedback on the ﬁndings? | p. 7, LN 15-16 |
| *Reporting* |  |  |
| 29. Quotations presented | Were participant quotations presented to illustrate the themes/ﬁndings? Was each quotation identiﬁed? e.g. participant number | Yes, 27 times in the capture Results, e.g. p. 12, LN 5-8 |
| 30. Data and ﬁndings consistent | Was there consistency between the data presented and the ﬁndings? | p. 7, LN 11-13 |
| 31. Clarity of major themes | Were major themes clearly presented in the ﬁndings? | We have attempted to present our findings in this work clearly in a manner that was consistent with the data collected. |
| 32. Clarity of minor themes | Is there a description of diverse cases or discussion of minor themes? |

**Once you have completed this checklist, please save a copy and upload it as part of your submission. Please DO NOT** **includes this checklist as part of the main manuscript document. It must be uploaded as a separate file.**
