# Supplementary material for: A qualitative study on the role of cultural background in patients' perspectives on rehabilitation
Source: BMC Musculoskelet Disord. 2012 Jan 23;13:5. doi: 10.1186/1471-2474-13-5 (PMC3398320; doi:10.1186/1471-2474-13-5)
Supplement: Additional file 2 — Coding tree. [file 1471-2474-13-5-S2.DOCX]

Coding tree

| **Main category** | **Subcategory/ related Codes (Pat/HP)^[[1]](#footnote-1)^** |
| --- | --- |
| **F0_Sociodemographic aspects** | - age (Pat) - education: school (Pat) - education: job (professional education) (Pat) - actual/ former occupational activity (Pat) - civil status (Pat) - size of domestic home (Pat) - domicile (Pat) - duration of stay in Switzerland (Pat) - function in Rehabilitation clinic (HP) |
| **F1_management of back pain** | **F1_1 previous management of back pain**   - duration of acute back pain of back pain (Pat) - duration of chronic back pain (Pat) - treatment history in Switzerland (Pat) - treatment in home country (HP) - medical treatment (Pat) - recommendations of the rehabilitation clinic (Pat) - first inpatient treatment (Pat) - former disorders (Pat) - treatment good for back pain (Pat) - course of disease in detail (Pat) - medication rated as highly important (HP)   **F1_2 cause and meaning of back pain**   - causes of disorders/ pain (Pat) - impact of pain (Pat) - psychological/psychiatric treatment (Pat) - battle his/her pain (Pat) - sitting a long time causes pain (Pat) - psychological care (Pat) - impact of psychological factors (Pat) - predominance of imaging procedures (X-ray, MRI, etc.) (Pat) - knowledge of pain memory/ classification on pain scale (Pat) - self-diagnosis/self-concept related to causes of back pain (Pat) - other diagnosis than made by physician (Pat) - pain as central point (HP) - pain as something special (HP) - pain is taken seriously (HP) - differences among patients in the group (HP)   **F1_3 rehabilitation by activity and exercise**   - friends with back pain in Switzerland and in former Yugoslavia (Pat) - activity without weight reduces pain (Pat) - activity is good (Pat) - activity causes pain (Pat) - rapid walking/running causes pain (Pat) - cause for non-acceptance of activity training (Pat) - helpful therapies (Pat) - non-helpful therapies (Pat) - negative experiences with health professionals (Pat) - positive experiences with health professionals (Pat) - measures causing relief (Pat) - general differences in treatment between Switzerland and former Yugoslavia (Pat) (HP) - more medication in Switzerland than in former Yugoslavia (Pat) - cause for aggravation of pain (Pat) - less patients with back pain in former Yugoslavia (Pat) - missing comprehension for therapy contents (HP) - opposition to therapy (HP) - background information enhancing comprehension for function-centred therapy (HP) - group dynamic: positive and negative effects (HP) |
| **F2_function-centred rehabilitation** | **F2_1 patients’ expectations of treatment**   - treatment: good (Pat) - treatment: bad (Pat) - expectations regarding treatment (Pat) - fulfilment of expectations (Pat) - success of treatment (Pat) - satisfaction with treatment/ therapies (Pat) - causes of satisfaction (Pat) - causes of dissatisfaction (Pat) - feeling good after therapy (Pat) - criticism on treatment (Pat) - suggestions for improvement of treatment (Pat) (HP) - very great expectations of patients (HP)   **F2_2 patients’ coping strategies**   - strategies avoiding/reducing pain (Pat) - strongest pain (Pat) - self-constraint (Pat) - self-therapy (Pat) - self-training (Pat) - expression of pain (HP) - medication is reducing pain (Pat) - passive attitude (HP) - medication rated as highly important (HP)   **F2_3 patients’ assessment of treatment**   - professional satisfaction with therapists (Pat) - interpersonal satisfaction with therapists (Pat) - professional satisfaction with physicians (Pat) - interpersonal satisfaction with physicians (Pat) - professional competencies of therapists (Pat) - interpersonal competencies of therapists (Pat) - professional competencies of physicians (Pat) - interpersonal competencies of physicians (Pat) - contact with therapists (Pat) - contact with physicians (Pat) - contact with patients (HP) - contact with physicians rated as highly important (Pat) (HP) - white coat effect (HP) |
| **F3_goals of rehabilitation** | **F3_1 goals of patients and health professionals**   - patients’ objective of treatment (Pat) - health professionals’ objectives (Pat) - no objectives, neither own objectives nor objectives formulated by professionals at the clinic (Pat) - not being briefed about the objectives of rehabilitation (Pat) - missing goal orientation (HP) - drafting together with patients individual objectives (HP) - contradictory statements of patients and health professionals (HP) - employment is many patients’ objective (HP)   **F3_2 return to work**   - years of occupational activity (Pat) - description of occupational activity (Pat) - satisfaction with current/ former occupational activity (Pat) - dissatisfaction with current/ former occupational activity (Pat) - health disorders at work (Pat) - work lacking compensation (HP) - work plays a major role (HP) - difficult labour situation (HP) - occupational activity enhances self-confidence (HP) - no occupation – social isolation (HP) - willingness to work (Pat) - work ability (Pat) - friends working in spite of back pain (Pat) - own occupational activity in spite of back pain (Pat) - preconditions of returning to work (Pat) - previous attempts to return to work (Pat) - free of pain = healthy = work (HP) |
| **F4_communication** | **F4_1 language barriers**   - information on treatment (Pat) - linguistic comprehensibility of information (Pat) - comprehensibility of information with regards to content (Pat) - information on disease/ pain memory (Pat) - comprehensibility of information on disease/ pain memory with regards to content (Pat) - linguistic comprehensibility of information on disease/ pain memory (Pat) - gap of information (Pat) - rationale for information gap (Pat) - requests related to information (Pat) - description of information brokering (Pat) - requests related to information brokering (Pat) - general linguistic problems/ difficulties in communication with physicians/ therapists (Pat) - difficulties in communication between patients and health professionals, caused by language barriers (HP) - communication in mother tongue is helpful (HP)   **F4_2 talking at across purposes**   - diagnosis made by physicians (Pat) - physicians don’t understand patients/ disbelieve patients (Pat) - distance between physicians and patients (Pat) - feeling of not being taken seriously, no comprehension for each other (Pat) - physicians and patients talk at cross-purposes (Pat) - communication about pain is difficult (pain scale) (HP) |
| **F5_family** | **F5_1 family support**   - familial environment (Pat) - familial support (Pat) - domicile of friends and family (Pat) - separation from the own family at the rehabilitation clinic (Pat) - continuous week-end visits during inpatient rehabilitation therapy (Pat) - secondary gain from illness (HP) - missing personal responsibility (HP) - missing social interactions/ contacts (HP) - missing body awareness (HP)   **F5_2 differences of family support between women and men**   - double burden of women: 100% work, 100% family (Pat) - no courage to face life, depressions (Pat) - stringent role allocation (HP) - men as main earner (HP) - women show slightly more activity in therapy than men (HP) |
| **F6_barriers to rehabilitation** | **F6_1 psychological aspects**   - anxiety/ precariousness related to physical activity, therapy, aggravation or recurrence of back pain (Pat) - anxiety of patients related to muscular load (HP) - unwillingness of report own history of disease (Pat) - side effects of therapies (Pat) - ill = bed rest (HP) - patients are afraid of pain (HP) - general anxiety (HP)   **F6_2 financial concerns**   - general expectations regarding their situation in Switzerland (Pat) - better life in Switzerland (Pat) - worries about the future (Pat) (HP) - heavy financial pressure (HP) - negative life circumstances (HP) |

1. Pat = Code out of the empirical data of patients (related to subcategory)

   HP = Code out of the empirical data of health professionals (related to subcategory) [↑](#footnote-ref-1)
